# Transpedicular Corpectomy in Minimally Invasive Surgery for Metastatic Spinal Cord Compression: A Single-Center Series

**DOI:** 10.7759/cureus.70503

**Published:** 2024-09-30

**Authors:** Gervith Reyes Soto, Daniel Vega Moreno, Monica Serrano-Murillo, Carlos Castillo-Rangel, Alberto Gonzalez-Aguilar, José Rodrigo Meré Gómez, Pablo Isaac Garcìa Fuentes, Bernardo Cacho Diaz, Manuel de Jesus Encarnacion Ramirez, Vladimir Nikolenko, Tshiunza M Cherubin, Miguel Agustín Amador Hernández, Nicola Montemurro

**Affiliations:** 1 Neurosurgical Oncology, Mexico's National Institute of Cancer, Tlalpan, MEX; 2 Neurosurgery, Universidad Nacional Autónoma de México, Coyoacán, MEX; 3 Neuro-oncology, National Institute of Oncology, Mexico, MEX; 4 Neurosurgery, Servicio of the 1ro de Octubre Hospital of the Instituto de Seguridad y Servicios Sociales de los Trabajadores del Estado, Instituto Politécnico Nacional, Mexico City, MEX; 5 Neurointerventional Surgery, Hospital Universitario San Ignacio, Bogotá, COL; 6 Physical Medicine and Rehabilitation, Clínica de la Columna Instituto Nacional de Rehabilitación, Mexico City, MEX; 7 Neurological Surgery, Hospital San Juan de Dios Guatemala, Guatemala, GTM; 8 Neurological Surgery, Instituto Nacional de Cancerologia, Mexico City, MEX; 9 Neurological Surgery, Russian People's Friendship University, Moscow, RUS; 10 Human Anatomy and Histology, I.M. Sechenov First Moscow State Medical University of the Ministry of Health of the Russian Federation (Sechenov University), Moscow, RUS; 11 Neurosurgery, Clinique Ngaliema, Kinshasa, COD; 12 Orthopaedics, Hospital Central Militar - Traumatología y Ortopedia, Hospital General de Mexico Cirugía de Columna, Mexico City, MEX; 13 Neurosurgery, Azienda Ospedaliero Universitaria Pisana, Pisa, ITA

**Keywords:** corpectomy, metastatic spinal cord compression, minimally invasive surgery, spinal metastases, transpedicular corpectomy

## Abstract

Introduction

The role of separation surgery in managing symptomatic spinal metastases has been increasing in recent years, and it represents a crucial part of the definitive management of this condition.

Methods

We report on a series of seven patients treated at the National Cancer Institute in Mexico using minimally invasive approaches to perform transpedicular corpectomy. The goal was to obtain a margin of tumor-free tissue, enabling the completion of oncological treatment with radiotherapy.

Results

We collected data from six cases. The mean age was 61.2 years. Surgical outcomes were good in 83.3% of patients. Ranging from minimally invasive instrumentations to total or partial corpectomies, these procedures achieved their intended function of generating healthy neural tissue free of tumor. This ensures that the radiation gradient does not affect this tissue. No surgical complications were reported. The objective of these surgeries was to establish a radiotherapy or radiosurgery regimen as soon as possible, thereby improving patients' quality of life (QoL).

Conclusions

Low-cost transpedicular corpectomy via minimally invasive surgery (MIS) is a safe and effective method that meets the goals of separation surgery. However, prospective studies are needed to directly compare open techniques with minimally invasive methods.

## Introduction

Spinal metastases are a common complication in cancer patients, with post-mortem studies indicating their presence in up to 70% of cases [1]. The thoracic spine is the most frequently affected, followed by the lumbar spine in 70% and 50% of cases, respectively [2]. Spinal metastases can lead to spinal or nerve compression in approximately 10% of patients, manifesting as symptomatic lesions [3]. The clinical presentation of these lesions is highly variable, influenced by factors such as the location of the metastasis, the affected spinal levels, and the growth characteristics of the tumor. Notably, up to 90% of patients presenting with acute neurological deficits due to spinal metastases have a history of localized pain [4]. The prognosis for patients with spinal metastatic lesions varies widely, influenced by the nature of the primary tumor, the patient's functional status, and the specific spinal levels involved [5]. Survival can range from a few months to over a year, highlighting the complex and variable nature of this condition. Consequently, surgical treatment for spinal metastases is primarily palliative, focusing on pain relief and the restoration of neurological function [6]. Surgery is part of a broader therapeutic strategy that includes radiotherapy, systemic treatments, and, increasingly, radiosurgery [7].

A relatively recent advancement in the surgical management of spinal metastases is the concept of separation surgery. This technique aims for circumferential decompression of neural tissue, creating a safe margin between the spinal cord and the vertebral body [8]. This margin allows for the application of high-dose radiotherapy to treat the metastatic lesion without damaging the healthy neural structures. Separation surgery was first described in 2000 by Bilsky MH et al. [9], utilizing a transpedicular corpectomy technique combined with epidural release and vertebral body resection. However, these initial techniques often required posterior spinal fusion, which increased recovery time, hospital stay, and the risk of complications. The timing of radiotherapy initiation post-surgery is crucial for optimal patient outcomes. Ideally, radiotherapy should begin within 1-2 weeks following surgery to maximize therapeutic benefits and minimize tumor progression [10]. However, achieving this timeline can be challenging due to complications associated with extensive surgical approaches and prolonged recovery periods. These challenges underscore the need for less invasive surgical techniques that can facilitate quicker recovery and timely radiotherapy.

In this context, minimally invasive surgery (MIS) has gained attention as a viable alternative to traditional open surgeries. MIS techniques offer several advantages, including reduced blood loss, shorter hospital stays, decreased postoperative pain, and faster recovery times [11]. Among these techniques, minimally invasive transpedicular corpectomy (MITC) has emerged as an effective method for achieving the goals of separation surgery. MITC involves the resection of the posterior vertebral wall and other compressive bony elements through a small, tubular retractor system, allowing for decompression and stabilization of the vertebral column [12]. The use of methylmethacrylate for stabilization avoids the need for extensive posterior spinal instrumentation, further reducing the surgical burden on patients.

Several studies have demonstrated the efficacy of MITC in managing metastatic spinal cord compression. Tatsui CE et al. [13] reported significant improvements in local control rates and overall survival among patients treated with spinal laser interstitial thermal therapy (LITT) and minimally invasive separation surgery followed by high-dose radiotherapy. Additionally, comparative studies have shown that MITC provides outcomes comparable to or better than traditional open surgeries, with fewer complications and quicker recovery times [14]. These findings suggest that MITC can effectively achieve the dual goals of decompression and stabilization while enabling early postoperative radiotherapy.

Despite these promising results, the adoption of MITC in clinical practice is not without challenges. The technique requires a high degree of surgical expertise and familiarity with minimally invasive spinal procedures. Moreover, patient selection is critical, as the success of MITC depends on factors such as tumor location, spinal stability, and the overall health status of the patient [15]. These considerations highlight the importance of multidisciplinary collaboration in the management of metastatic spinal lesions.

Our study aims to contribute to the growing body of evidence supporting the use of MITC for metastatic spinal cord compression. We present a case series of seven patients treated at the National Cancer Institute in Mexico, focusing on the surgical technique, postoperative outcomes, and subsequent radiotherapy regimens. This series underscores the feasibility and effectiveness of MITC in achieving the objectives of spinal cord decompression, including separation surgery for radiosurgery, particularly in a resource-limited setting.

The primary goal of this study is to demonstrate that MITC can achieve effective decompression and stabilization, enabling the early initiation of radiotherapy and improving patient outcomes. Additionally, we aim to provide a detailed account of the surgical technique and perioperative management to aid in the dissemination and adoption of this approach in other clinical settings.

## Materials and methods

Study design and setting

This retrospective study was conducted at the National Cancer Institute in Mexico, focusing on a case series of six patients who underwent MITC for metastatic spinal cord compression. The study was conducted in accordance with the Declaration of Helsinki and was approved by the Ethics Committee of Instituto Nacional de Cancerología, Mexico City, Mexico (Jan/2024). All patients provided informed consent.

Patient selection and preoperative assessment

Patients were selected based on the following criteria: 1) diagnosis of symptomatic metastatic spinal cord compression, as well as bone metastases from multiple myeloma; 2) indications for separation surgery as determined by a multidisciplinary tumor board, including oncologists, radiologists, and spine surgeons; 3) lesions amenable to minimally invasive approaches; 4) exclusion of patients with significant medical comorbidities that precluded surgery.

Patients underwent a comprehensive preoperative evaluation, including clinical assessment, radiological evaluation, and scoring systems: Spinal Instability Neoplastic Score (SINS) and Neurological, Oncological, Mechanical, and Systemic (NOMS).

Surgical technique

Patients were placed under general anesthesia and positioned prone on a radiolucent operating table with proper padding to avoid pressure points. A minimally invasive tubular transpedicular approach was employed:

Incision

A 1.5 cm paramedian (3 cm from midline) incision was made at the pre-marked level based on fluoroscopic guidance.

Tubular Retractor Placement

A 20 cc syringe cut at the proximal end was utilized as a tubular port, facilitating muscle and soft tissue dissection down to the lamino-transverse and lamino-facet junction.

Pedicle Drilling

The pedicle of the affected vertebra was drilled to access the posterior vertebral body.

Corpectomy

The posterior component of the vertebral body was resected using high-speed burrs and rongeurs. Care was taken to fully decompress the spinal canal.

Cementoplasty

The drilled cavity was filled with methylmethacrylate to restore vertebral body height and provide structural support, according to intraoperative radiographic findings. Patients were monitored in the recovery unit with standard postoperative protocols, including pain management, neurological assessments, and early mobilization within 24 hours post-surgery.

Radiotherapy

Radiotherapy was planned to commence as early as possible post-surgery, typically within 1-2 weeks. The dosing varied based on individual cases and tumor histology, ranging from 15 Gy in 5 fractions to 30 Gy in 10 fractions, according to our protocols.

Outcomes measured

At discharge, neurological improvement was assessed using the Frankel grading system. Postoperative pain was assessed using the Visual Analog Scale (VAS). For radiological outcomes, postoperative MRI scans were analyzed for spinal cord decompression and vertebral body stability. Any intraoperative or postoperative complications were recorded. Survival and quality of life (QoL) were followed up to six months post-surgery, using the WHO Quality of Life (WHOQOL-100) scale.

## Results

We collected a total of 6 cases. The mean age was 61.2 years old. Table 1 shows all details.

**Table 1:** Patient demographics and study outcomes. NR: Not rated; RT: Radiation therapy; VAS: Visual Analogue Scale.

Patient No.	Sex/Age	Level surgically treated	Primary cancer type	Preoperative symptoms	Time to Surgery to First Dose of RT (week)	Neurological outcome at discharge (Frankel Score)	Postoperative pain (VAS)	Complications	Survival and quality of life
1	F/68	T11	Luminal A breast cancer	Conus medullaris syndrome	2	D	2	None	Improved
2	F/63	T5	Papillary thyroid cancer	Anterior spinal cord syndrome	3	D	3	None	Improved
3	F/56	T1-T2	Luminal A breast cancer	Axial pain	6	E	1	None	Improved
4	F/65	T8	Multiple myeloma	Anterior spinal cord syndrome	NR	D	3	None	Improved
5	F/62	L2	Multiple myeloma	Axial pain	NR	D	2	None	Stable
6	F/53	T6 and T9	Lung adenocarcinoma	Complete spinal cord syndrome	2	C	4	None	Worsed (6 months post-surgery)

Case 1

**Figure 1:**
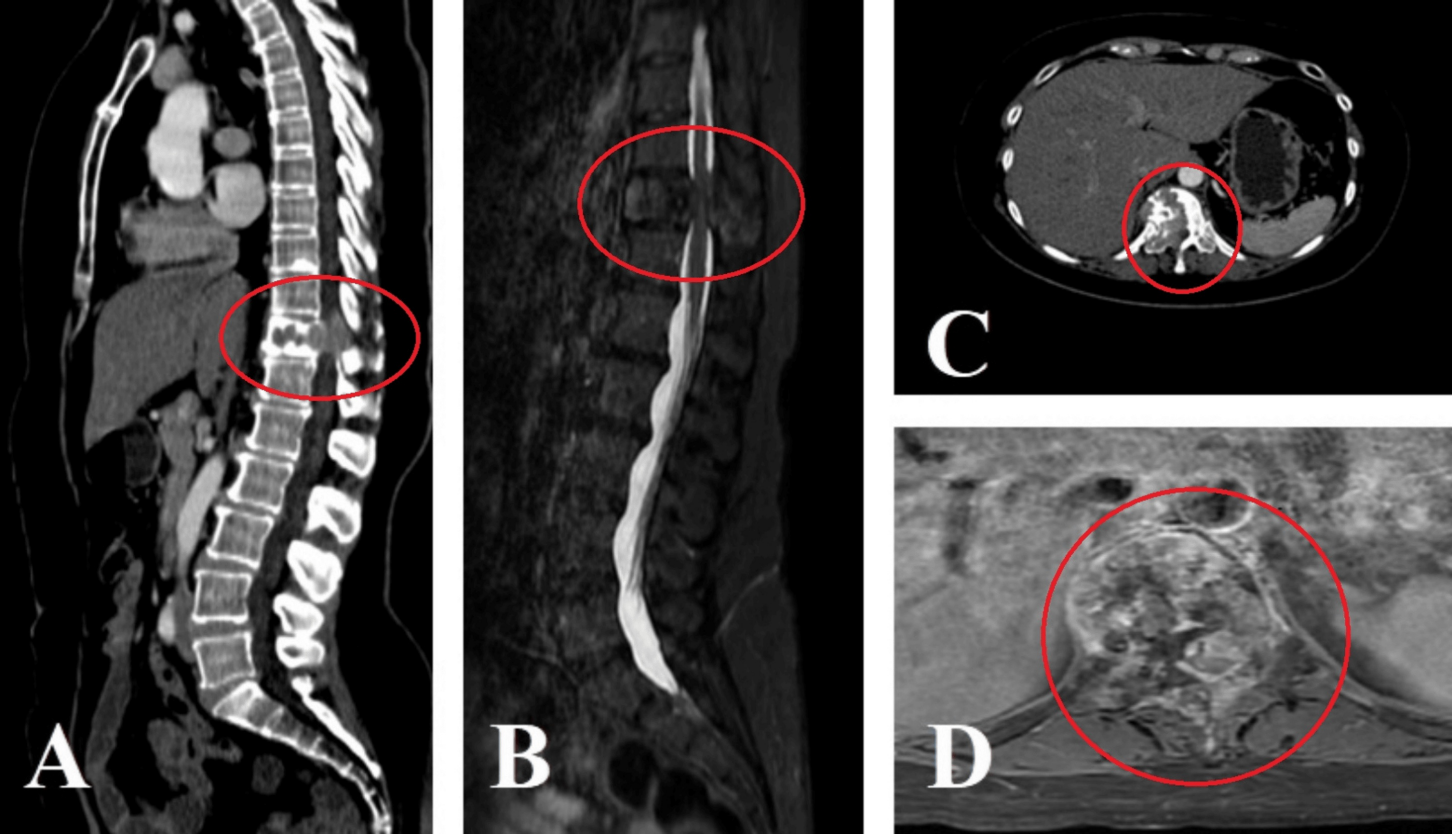
(A) Computed tomography (CT) scan of the spine shows a T11 pathological compression fracture with displacement of the posterior wall and invasion of the spinal canal. (B) MRI scan of the spine shows compression of the spinal canal due to a metastatic lesion. (C and D) CT and MRI axial sections show a lytic lesion of the vertebral body of T11, facets, and right pedicle, as well as the lamina and spinous process.

A 68-year-old female patient diagnosed with breast cancer in 2008, ER, PR+, HER2-. Ten years after being under control, she presented with sudden axial pain, urinary difficulty, and weakness in both lower limbs. She was taken to the emergency room for evaluation (Figure 1). A compression fracture of the T11 vertebra with blastic and lytic characteristics was found, involving the right facet and invading more than 50% of the spinal canal. Evaluation based on the SINS scale yielded a score of 8, categorized as indeterminate stability. Using the NOMS classification, she was a candidate for decompressive surgery to treat compressive myelopathy without instrumentation due to a moderately radiosensitive tumor, good functional status, myelopathy, and previous single-dose radiotherapy of 8 Gy.

**Figure 2:**
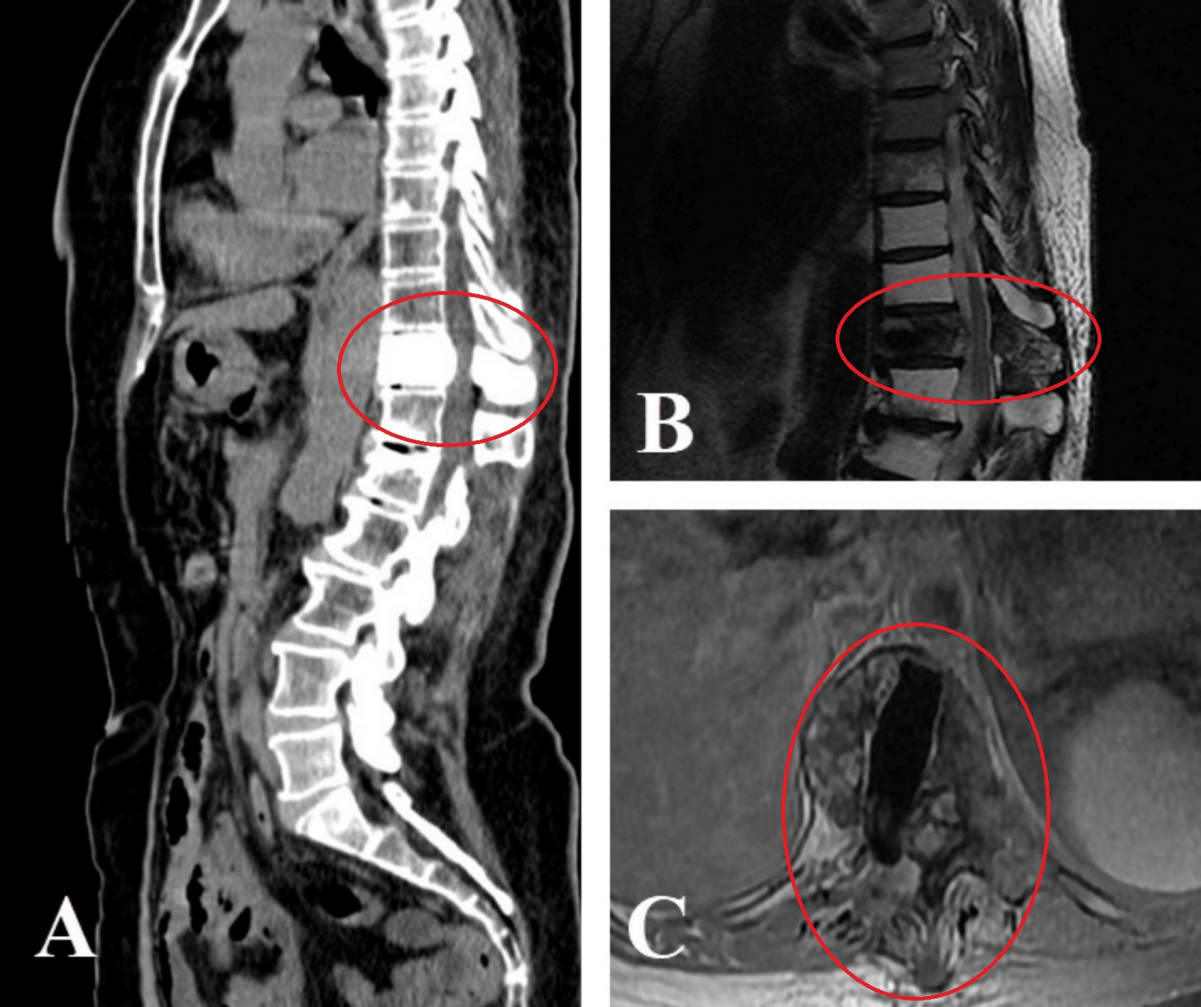
(A) CT scan of the spine shows vertebral augmentation of T11 with methylmethacrylate, preservation of sagittal balance, and decompression of the spinal canal. (B) MRI scan shows passage of cerebrospinal fluid through T11, with T11 appearing hypointense, corresponding to resected bone tissue replaced by methylmethacrylate. (C) Axial section shows the corpectomy site with its path and the area replaced with synthetic material.

**Figure 3:**
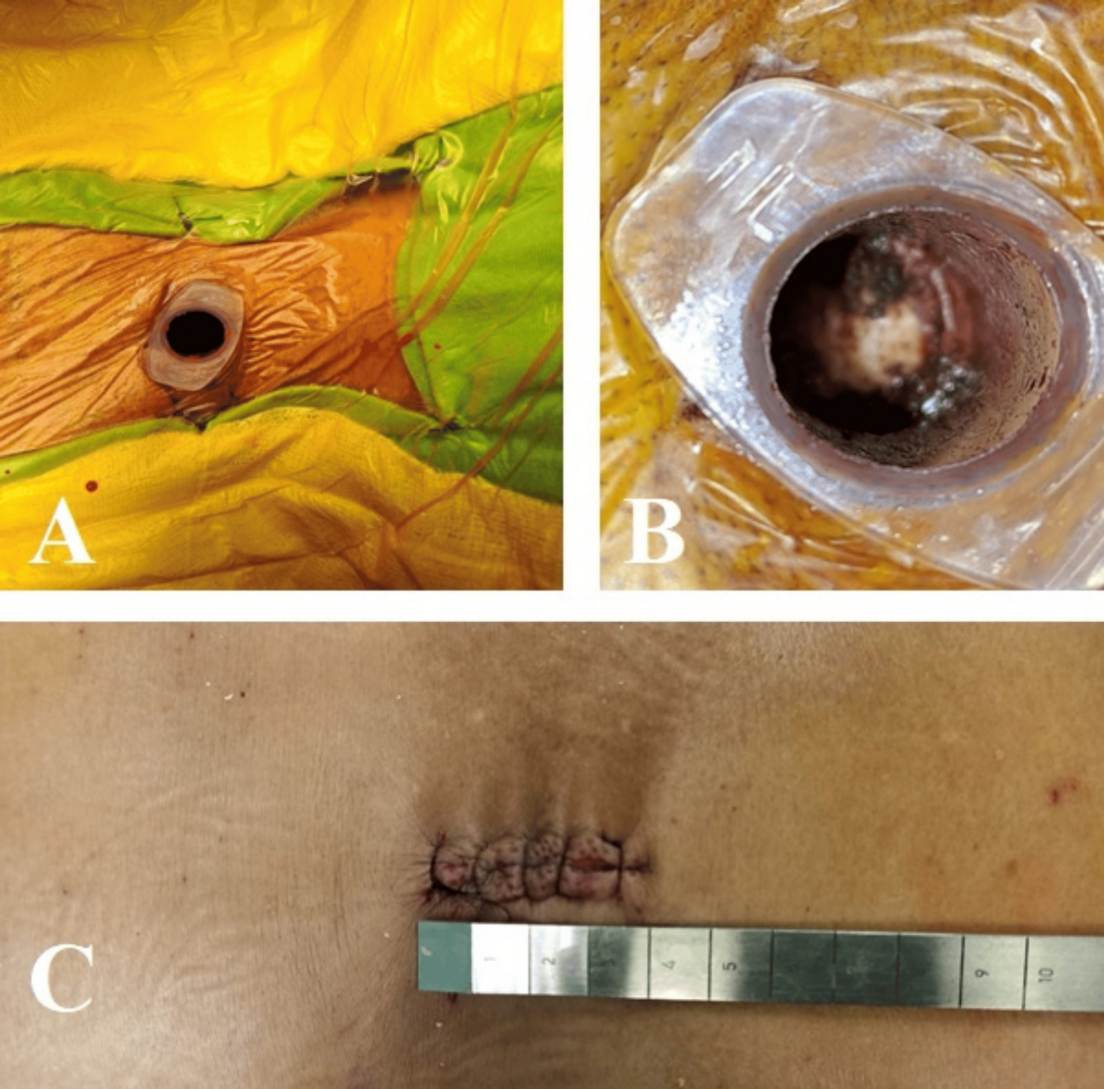
Single port for MIS using a 20 milliliter syringe (A, B) as a tubular port, inserted at the lamino-transverse junction. (C) Skin incision measuring just 3.5 cm. MIS: Minimally Invasive Surgery.

A 1.5 cm incision was made, pre-marked with fluoroscopy over the lamino-transverse and lamino-facet junction to locate the T11 vertebral pedicle. A 20 cc syringe was used as a tubular port. Muscle and soft tissue dissection was performed. The intersection point between the facet and the transverse process was located, the tubular port was placed, and drilling was performed on the right T11 pedicle. The posterior component of the vertebral body was resected, the nerve root was identified, particularly its shoulder, and decompression of the canal was verified using a pedicle feeler. Once decompression was confirmed, the previously drilled space was filled with methylmethacrylate, preserving the vertebral body's height and increasing the construct to prevent collapse and subsequent fractures. As it was a single level, with no loss of sagittal or coronal balance and structural support provided by the cementoplasty, posterior fixation was avoided, thus preventing a larger surgery with higher complication risks (Figures 2-3). The patient recovered satisfactorily, was discharged two days post-surgery, and received adjuvant radiotherapy two weeks later at a dose of 30 Gy in 10 fractions.

Case 2

**Figure 4:**
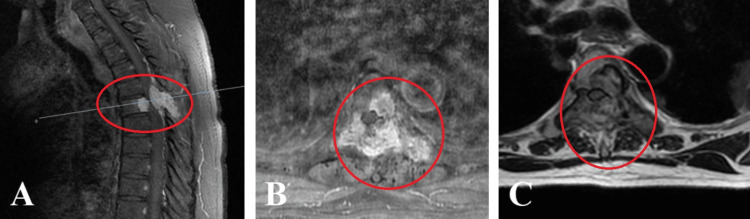
Sagittal (A) and axial (B, C) MRI scans of the thoracic spine show a metastatic tumor at T5, involving the vertebral body, a portion of the left posterior wall, both pedicles, laminae, and spinalis muscles, and invading the spinal canal with compression of the same. There is an absence of CSF passage.

**Figure 5:**
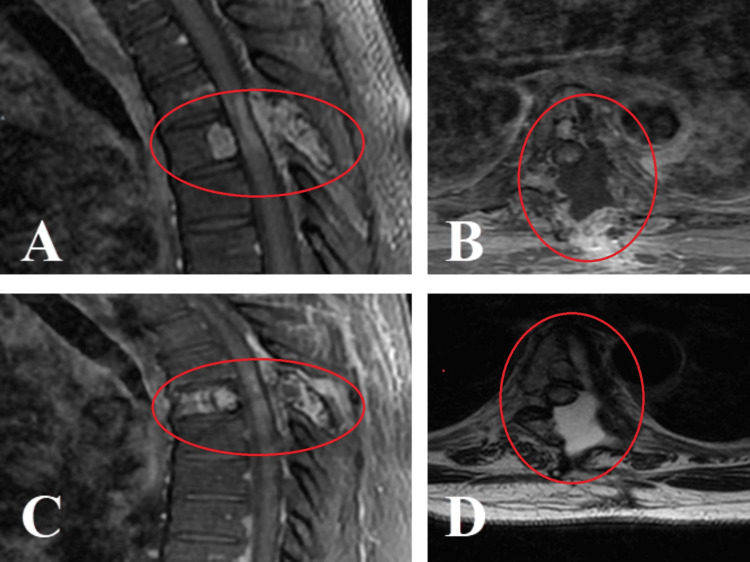
Post-operative MRI scan (A and B) shows a hypointense area that corresponds to the third project of circumferential decompression performed using MIS. Post-radiotherapy MRI scan (C and D) shows improvement in the width of the spinal canal and resolution of spinal cord compression. MIS: Minimally Invasive Surgery.

A 63-year-old female with a history of papillary thyroid cancer and multiple bone metastases to the appendicular skeleton presented with complete spinal syndrome in October 2018, treated with a single 8 Gy dose of radiotherapy without symptom improvement. She persisted with lower limb weakness, sphincter alterations, and a T3-T4 sensory level. She was classified with a SINS score of 8 (indeterminate stability) and under the NOMS classification as a patient with a radio-resistant tumor, previous radiotherapy, myelopathy, and good functional status, thus opting for separation surgery. The same procedure described previously was performed, with a transpedicular T5 left approach. The patient was discharged 48 hours post-surgery, improved to a Frankel D, and received a second cycle of conventional external beam radiation three weeks later at a dose of 30 Gy in 10 fractions (Figures 4-5).

Case 6

**Figure 6:**
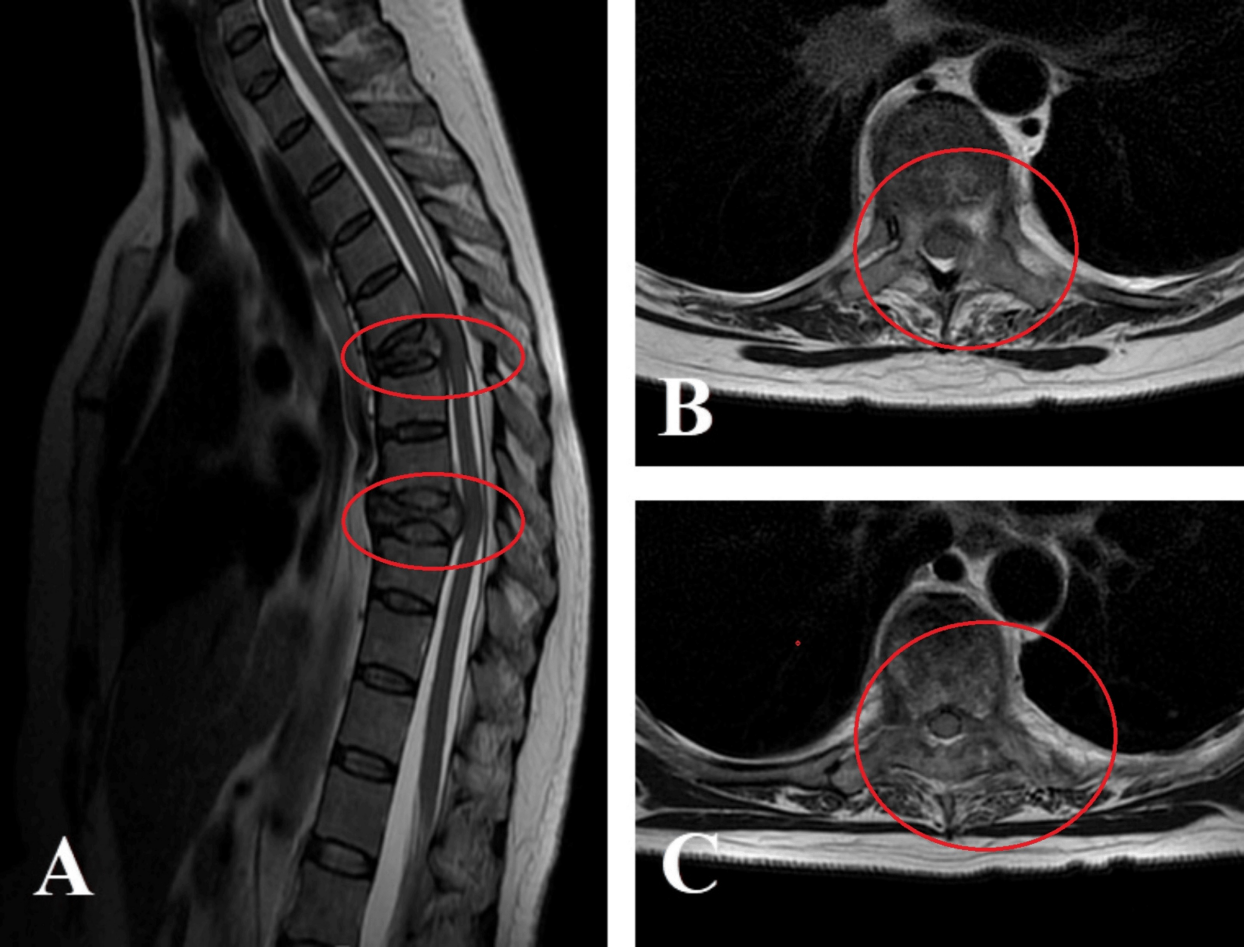
Sagittal (A) and axial (B, C) MRI scans of the thoracic spine show metastatic lesions at T6 and T9 with spinal cord compression, loss of more than 50% of body height, and invasion into the spinal canal. Mainly, ventral compression of the spinal cord is observed.

**Figure 7:**
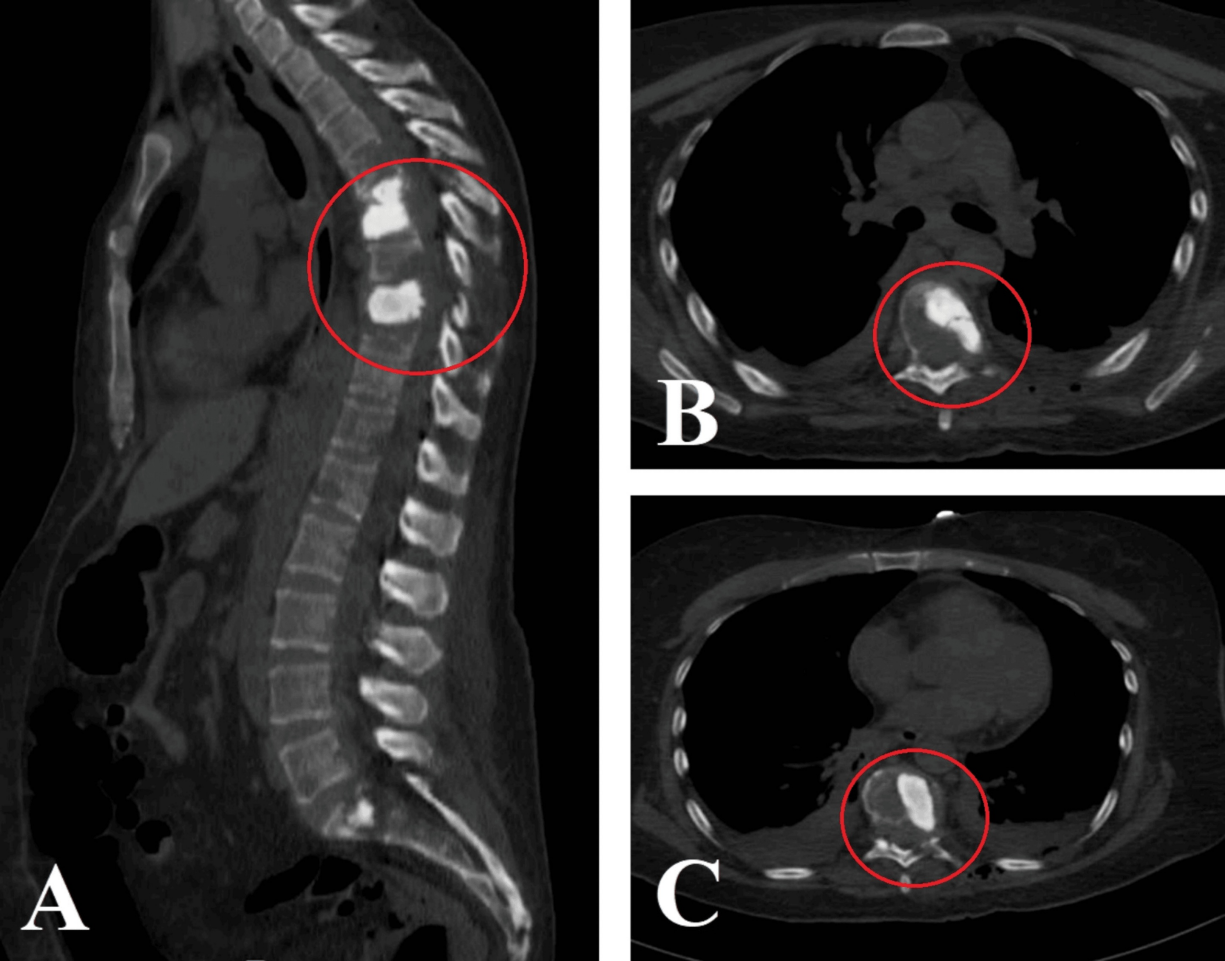
Sagittal (A) and axial (B, C) CT scans of the thoracic spine show vertebral augmentation of the T6 and T9 vertebral bodies with methylmethacrylate, corpectomy of the posterior walls of these vertebrae, and an increase in the space of the medullary canal.

A 53-year-old female was diagnosed with lung adenocarcinoma with bone activity. In October 2022, she presented with axial pain and sudden bilateral leg weakness. Imaging showed metastatic lesions to the thoracic spine at T6 and T9. She scored 12 on the SINS scale, categorized as having indeterminate stability, and was identified as a NOMS candidate for separation surgery without fixation. The same approach as previously described was used. A low-cost tubular approach for hemicorporectomy of the T6 and T9 vertebral bodies was performed in a single surgical session. Two weeks later, the patient received radiotherapy at T6 and T9 at a dose of 20 Gy in 5 sessions. She showed significant functional recovery over the following two months, but due to an uncontrolled primary tumor, she died six months after surgery from pneumonia (Figures 6-7).

## Discussion

While spondylectomies can be considered part of the management for patients with vertebral fractures secondary to metastatic lesions, they are not always feasible in the context of oncological patients [16]. The placement of transpedicular cages for traumatic pathologies in the thoracic spine has been described, where recovery days can be longer. Sometimes, interbody cage placement via transpedicular approaches has also been performed in the lumbar spine, preserving roots, albeit with extensive approaches requiring longer postoperative recovery times [17-19].

One indication for a MIS approach is unilateral facet or pedicle involvement. We demonstrated that even with bilateral involvement, as in the second presented case with bilateral compression and facet involvement, effective circumferential decompression was possible through the same port. Although percutaneous pedicle screw fixations and decompressions through small incisions have been well described, they are reserved for cases of significant instability, with loss of sagittal or coronal balance [20,21]. While these techniques yield excellent results, recovery times are longer compared to single-port procedures that do not require screw fixation [22]. Corpectomy or spondylectomy for metastatic disease has evolved over the years. Traditional placement of cages through minimally invasive approaches has been described, but these carry risks of subsidence, migration, fracture, infections, longer hospital stays, among other complications [23-25]. In oncological patients requiring early initiation of radiotherapy, such events must be avoided [26].

Separation surgery has become a critical intervention in managing metastatic spinal cord compression. The primary objective is to achieve circumferential decompression of neural tissue, creating a safe margin between the tumor and the spinal cord [27,28]. This facilitates the effective application of radiosurgery without damaging healthy neural structures. Initially described by Bilsky MH et al. in 2000, this concept has revolutionized the approach to metastatic spinal lesions by emphasizing decompression and stabilization to facilitate prompt radiotherapy [9,10,28]. Bilsky’s technique involves a single-stage posterolateral transpedicular approach for spondylectomy, epidural decompression, and circumferential fusion, and remains a cornerstone in the management of spinal metastases. However, the extensive nature of this surgery often necessitates longer recovery periods and delays the initiation of adjuvant therapies. Our minimally invasive method offers a viable alternative, particularly in patients where rapid recovery and early radiotherapy are critical [9,10].

Our series of seven patients treated with MITC aligns with the growing evidence supporting minimally invasive techniques for spinal metastases [29,30]. The efficacy of MITC in providing immediate decompression, restoring vertebral stability, and enabling early radiotherapy is well-documented [31]. For instance, Hong SH et al. demonstrated that separation surgery significantly improves local disease control and patient outcomes when followed by adjuvant hypofractionated or high-dose single-fraction stereotactic radiosurgery [32]. Minimally invasive spinal surgery (MISS) offers several advantages over traditional open techniques, including reduced intraoperative blood loss, shorter hospital stays, decreased postoperative pain, and faster recovery times. Ahmed AK et al. conducted a comparative study between minimally invasive spine surgery and traditional open surgery for spinal metastasis, finding that MISS provided comparable or superior outcomes in terms of neurological improvement and pain relief, with fewer complications and shorter recovery periods [33,34]. Our findings are consistent with this, showing good outcomes (83%) and pain relief with minimal complications in our series.

The corpectomy proposed in our series involves the resection of the bony tissue, mainly the posterior vertebral wall but not the entire body, meaning only the bone fragment displaced towards the spinal canal is removed [35]. This creates enough space to apply radiotherapy without the risk of myelopathy or neuropathy. Additionally, the application of methylmethacrylate provides support to the vertebral body, reducing the risk of fractures or instability [36,37]. Circumferential separation surgery via MIS has proven to be one of the standard treatments for managing metastatic spinal lesions, even showing additional benefits compared to open approaches [22,36,38]. In our series, the use of a 20 cc syringe as a tubular port for the transpedicular approach exemplifies a cost-effective and innovative method for achieving the goals of separation surgery. This technique allows for precise decompression and the application of methylmethacrylate to restore vertebral body height and stability [39,40]. Traditional approaches, such as those involving the placement of expandable cages, carry risks of subsidence, migration, and infection, which are mitigated by our method [41,42]. Our approach also avoids the need for extensive posterior spinal fusion, which has been associated with increased recovery time and higher complication rates. This is particularly relevant in oncological patients, where rapid initiation of adjuvant therapies is critical for improving overall prognosis [43]. Eleraky M et al. [44] reported that traditional open approaches requiring posterior fusion had longer recovery times and delayed the start of radiotherapy [45].

Case-specific outcomes

Case 1 featured a 68-year-old female with a history of breast cancer and a compression fracture at T11. The minimally invasive approach allowed for effective decompression and stabilization, enabling the patient to receive radiotherapy within two weeks post-surgery. This prompt initiation of adjuvant therapy aligns with findings by Tseng et al. [46], who emphasized the importance of early radiotherapy in improving local control and survival outcomes in spinal metastases.

Case 2 involved a 63-year-old female with papillary thyroid cancer who underwent T5 transpedicular corpectomy. Postoperative recovery was smooth, and the patient received 30 Gy in 10 fractions of radiotherapy. This case highlights the effectiveness of our technique in managing radio-resistant tumors, as also supported by the work of Saigal R et al. [16], who stressed the necessity of combining surgery with radiotherapy in managing resistant metastatic lesions.

Case 6 featured a 53-year-old female with lung adenocarcinoma and metastatic lesions at T6 and T9. Despite the advanced disease, the patient showed significant functional recovery post-surgery. However, she succumbed to complications related to her primary tumor six months later. This underscores the complexity of treating metastatic spinal disease and the need for multidisciplinary management, as discussed by Nathan SS et al. [47]. The adoption of minimally invasive techniques in spinal surgery has significant implications for clinical practice. These techniques not only reduce surgical morbidity but also enable rapid recovery and early initiation of adjuvant therapies, improving overall patient outcomes [16,48,49]. Our series demonstrates that even in cases with bilateral involvement, effective circumferential decompression can be achieved through a single port, highlighting the versatility and effectiveness of minimally invasive approaches [50,51].

Challenges and future directions

The advent of minimally invasive surgery (MIS) has revolutionized the management of metastatic spinal cord compression. With the integration of advanced technologies such as 3D printing, augmented reality (AR), exoscopes, and molecular treatments, the future of spinal surgery is poised for significant advancements.

3D printing technology offers the potential for patient-specific surgical planning and the creation of customized implants. In the context of transpedicular corpectomy, 3D-printed models of the spine can be used to simulate surgeries, allowing for precise preoperative planning [52-54]. These models enable surgeons to visualize complex anatomical structures and practice the surgical approach, thus reducing intraoperative time and improving outcomes [55]. Customized 3D-printed implants can be designed to fit the unique anatomical contours of a patient's vertebral column, providing enhanced stability and reducing the risk of postoperative complications [55-57].

AR technology can significantly enhance the precision of minimally invasive spinal surgeries. By overlaying digital images onto the surgeon’s field of view, AR provides real-time guidance during surgery [58]. This technology allows for the visualization of critical structures such as nerves and blood vessels, minimizing the risk of injury. In transpedicular corpectomy, AR can assist in accurate pedicle screw placement and the resection of metastatic lesions, ensuring thorough decompression of the spinal cord. The use of AR can also facilitate the training of surgeons, providing immersive and interactive learning experiences [59-61].

Exoscopes are high-definition digital visualization tools that offer magnified views of the surgical field [62]. Unlike traditional microscopes, exoscopes provide a broader field of view and greater flexibility in positioning. In transpedicular corpectomy, exoscopes enhance the surgeon’s ability to perform precise and delicate maneuvers within the confined space of the spinal canal [63,64]. The improved visualization helps in achieving complete tumor resection and effective spinal decompression. Furthermore, exoscopes can be used in conjunction with AR to provide an integrated surgical navigation system [65,66].

Advances in molecular biology have led to the development of targeted therapies that can be used in conjunction with surgical interventions [67]. Molecular treatments aim to inhibit specific pathways involved in tumor growth and metastasis. In the context of spinal metastases, targeted therapies can be administered preoperatively to shrink tumors, making them more amenable to surgical resection [67]. Postoperatively, these therapies can help in controlling residual disease and preventing recurrence [68]. The combination of molecular treatments with minimally invasive surgery holds the promise of improving long-term outcomes for patients with metastatic spinal cord compression [69-71].

Limitations of the study

One of the primary limitations of this study is the small sample size, consisting of only seven patients who underwent surgery in a single center. This limited cohort restricts the generalizability of our findings to a broader population of patients with metastatic spinal cord compression. Larger, multicenter studies are necessary to validate our results and establish more robust evidence for the effectiveness and safety of MITC. In addition, the retrospective design of this study inherently carries certain biases and limitations. Retrospective studies are often subject to selection bias, as the inclusion criteria and patient selection may not represent the entire spectrum of patients with metastatic spinal lesions. Additionally, the accuracy and completeness of the collected data can be influenced by the quality of medical records and the recall bias of the clinicians involved.

This study lacks a control group of patients treated with traditional open surgical techniques. As a result, direct comparisons between minimally invasive and open approaches cannot be made. Future prospective studies with randomized controlled trials are needed to provide more definitive evidence on the comparative effectiveness and safety of these surgical techniques. The follow-up period in this study was limited to a minimum of six months post-surgery. This short follow-up period may not fully capture the long-term outcomes and potential late complications associated with MITC. Longer follow-up is necessary to assess the durability of the surgical results, the risk of recurrent spinal metastases, and the overall survival of the patients.

The heterogeneity of the primary tumors and their metastatic locations presents another limitation. The seven patients included in this study had various primary cancers and spinal involvement at different levels. This variability can affect the generalizability of the results, as different tumor types and locations may respond differently to the same surgical intervention. While radiotherapy is a crucial component of the treatment strategy for metastatic spinal cord compression, our study includes limited data on the specifics of radiotherapy protocols and their outcomes. A detailed analysis of radiotherapy dosing, techniques, and timing in conjunction with MITC would provide a more comprehensive understanding of the overall treatment efficacy.

QoL assessments were not included in this study. QoL is an essential outcome measure, particularly in palliative care settings. Future studies should incorporate standardized QoL assessments to evaluate the impact of MITC on patients' overall well-being, functional status, and pain management. The technical expertise required for MISS and the associated learning curve were not addressed in this study. The success and complication rates of MITC may vary depending on the surgeon's experience and familiarity with the technique. Future research should consider the impact of the learning curve on surgical outcomes and explore strategies to optimize training and proficiency in MITC.

Potential confounding variables, such as variations in preoperative functional status, comorbidities, and adjuvant treatments, were not controlled for in this study. These factors could influence the surgical outcomes and need to be accounted for in future prospective studies to ensure a more accurate assessment of the efficacy and safety of MITC.

Finally, while our study provides valuable insights into the feasibility and effectiveness of MITC, prospective studies are necessary to confirm these findings. Randomized controlled trials with larger sample sizes, standardized protocols, and long-term follow-up will help establish MITC as a reliable option for the management of metastatic spinal cord compression.

## Conclusions

We present a series of seven patients treated at a specialized center for complex spinal tumor pathology using MIS. Separation surgeries were performed to ensure the patients could recover as quickly as possible, continue their oncological treatment, and substantially improve their functionality. We avoided large surgeries involving posterior fusion in all cases due to the high risk of complications and the need for rapid initiation of radiotherapy. Transpedicular corpectomy with cementoplasty is a good therapeutic strategy for two main objectives: achieving separation surgery that can be complemented with radiotherapy as quickly as possible, and using cementoplasty to prevent subsequent fractures, remove diseased bone, and reduce the characteristic osteogenic postoperative pain of metastatic lesions. This approach also avoids the use of more expensive osteosynthesis material that could increase patient comorbidities.
